# Silicon-On-Sapphire Metasurfaces Generate Arrays of Dark and Bright Traps for Neutral Atoms

**DOI:** 10.1021/acsphotonics.6c01311

**Published:** 2026-07-06

**Authors:** Chengyu Fang, Minjeong Kim, Hongyan Mei, Xuting Yang, Zhaoning Yu, Yuzhe Xiao, Sanket Deshpande, Preston Huft, Alan M. Dibos, David A. Czaplewski, Mark Saffman, Jennifer T. Choy, Mikhail A. Kats

**Affiliations:** † Department of Electrical and Computer Engineering, 5228 University of Wisconsin–Madison, 1415 Engineering Drive, Madison, Wisconsin 53706, United States; ‡ Department of Materials Science and Engineering, University of Wisconsin–Madison, 1509 University Avenue, Madison, Wisconsin 53706, United States; § Department of Physics, University of North Texas, 210 Avenue A, Denton, Texas 76201, United States; ∥ Department of Physics, University of Wisconsin–Madison, 1150 University Avenue, Madison, Wisconsin 53706, United States; ⊥ Center for Nanoscale Materials, 1291 Argonne National Laboratory, Lemont, Illinois 60439, United States; # Infleqtion, Madison, Wisconsin 53703, United States

**Keywords:** optical metasurface, optical tweezer, bottle beam, atom array, atom trap, dark trap, silicon, Gerchberg-Saxton

## Abstract

We demonstrated crystalline silicon-on-sapphire (c-SOS) metasurfaces that convert a Gaussian beam into arrays of complex optical traps, including arrays of optical bottle beams that trap atoms in dark regions interleaved with bright tweezer arrays. The high refractive index and indirect band gap of crystalline silicon make it possible to design high-resolution near-infrared (λ > 700 nm) metasurfaces that can be manufactured at scale using CMOS-compatible processes. Compared with active components like spatial light modulators (SLMs) that have become widely used to generate trap arrays, metasurfaces provide an indefinitely scalable number of pixels, enabling large arrays of complex traps in a very small form factor, as well as reduced dynamic noise. To design metasurfaces that can generate three-dimensional bottle beams to serve as dark traps, we modified the Gerchberg-Saxton algorithm to enforce complex-amplitude profiles at the focal plane of the metasurface and to optimize the uniformity of the traps across the array. We fabricated and measured c-SOS metasurfaces that convert a Gaussian laser beam into arrays of bright traps, dark traps, and interleaved bright/dark traps.

## Graphical abstract

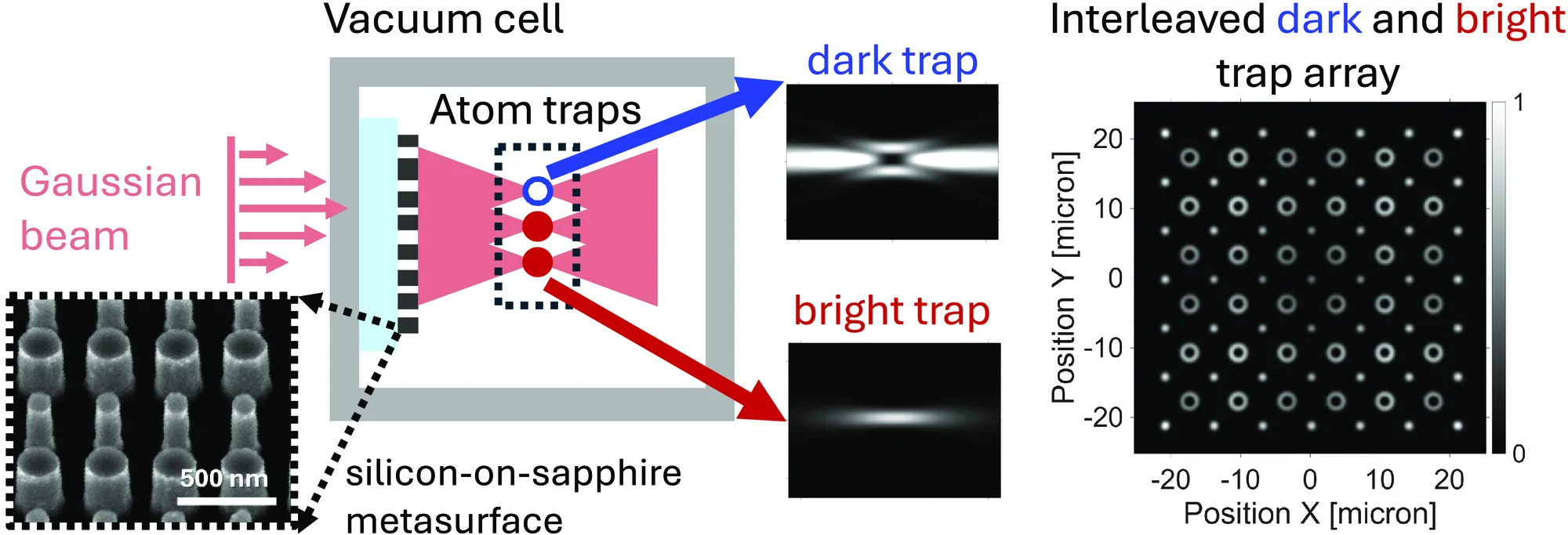

## Introduction

The trapped-neutral-atom array is an emerging platform for quantum information processing, quantum sensing, and quantum communications.^1^ Arrays of trapped neutral atoms are typically realized using optical tweezers generated by active optical elements, including spatial light modulators (SLMs),^2–5^ acousto-optic deflectors (AODs),^6–8^ and digital micromirror devices (DMDs).^9,10^ While flexible and powerful, these optical devices face several intrinsic limitations. The limited number of pixels (in SLMs and DMDs) or RF tones (in AODs) constrains the upper limit on the number of optical tweezers that can be generated simultaneously; for instance, neither an N × N DMD nor an N × N SLM can realize an N × N tweezer array because forming the appropriate field distribution around each trap requires multiple degrees of freedom (i.e., pixels in an SLM or DMD), so the system supports far fewer than *N* ^2^ traps. Also, the large pixel pitch (typically several microns) relative to the trapping laser wavelength (∼ 700–1100 nm) means that demagnification is required to generate diffraction-limited optical traps, complicating the optical setup. Active components can also introduce dynamic noise,^11^ which compromises the stability of quantum systems.^12^

An alternative approach is to use passive optical components, for example, amplitude masks with a spatial filter and imaging optics that have been used to produce single- and dual-species arrays,^13,14^ or optical metasurfaces that offer subwavelength phase control through large arrays of engineered nanostructures.^15–19^ In other contexts, metasurfaces comprising more than ten billion elements (i.e., pixels) have been demonstrated,^20^ suggesting a route for the generation of very large atomic arrays with traps of arbitrary complexity using this technology. To date, however, metasurface demonstrations for atom trapping have been limited to the generation of optical-tweezer arrays.^15,16,18,19^

Here, we demonstrate crystalline silicon-on-sapphire (c-SOS) metasurfaces that convert a Gaussian input beam into arrays of complex optical traps: bottle-beam (dark trap) arrays, tweezer (bright trap) arrays, and interleaved bottle-beam and tweezer arrays. Among these, bottle beams are challenging to generate but offer several advantages for quantum applications over optical tweezers: confining atoms at an intensity null reduces photon scattering and enables longer trapping lifetime,^21,22^ and the small local intensity at the atoms also makes the traps less sensitive to fluctuations of the trapping-laser power. To generate the sophisticated intensity profiles for bottle beams and optimize the uniformity across the array, we adapted a modified form of the Gerchberg-Saxton (G-S) algorithm^23^ that enforces both amplitude and phase in the image plane of the metasurface. The resulting phase profile was encoded into a c-SOS metasurface, where silicon’s high refractive index enables smaller pixel spacing^24^ compared to metasurfaces with lower-index materials. The resulting metasurfaces, fabricated with CMOS-compatible processes, were used to generate a 7 × 7 bottle-beam array, a 21 × 21 bright array, and an interleaved bright(7 × 7)/dark(6 × 6) array.

## Working Principle and Design

Our approach, shown in Figure 1, is to send a Gaussian beam through a c-SOS metasurface to form arrays of optical traps at the focal plane. Depending on the target application, the arrays can be configured to be bright tweezer arrays, dark bottle-beam arrays, interleaved bright/dark arrays, or some other desired arrangement. The metasurface can be positioned outside of the vacuum cell (Figure 1(A)) or inside the vacuum cell (Figure 1(B)). In the out-of-vacuum configuration, the trapping profile is first generated outside and then reimaged inside the cell. This approach offers high flexibility, allowing metasurfaces to be swapped without breaking the vacuum and incorporating functions (e.g., beam splitting for fluorescence readout) into the reimaging optics. Alternatively, in the in-vacuum configuration, the metasurface can be mounted directly inside the vacuum cell, for example, as part of the cell wall. This arrangement provides the best integration by eliminating the reimaging optics, the design of which can be nontrivial due to the need to compensate for the cell wall.^25^

**Figure 1:**
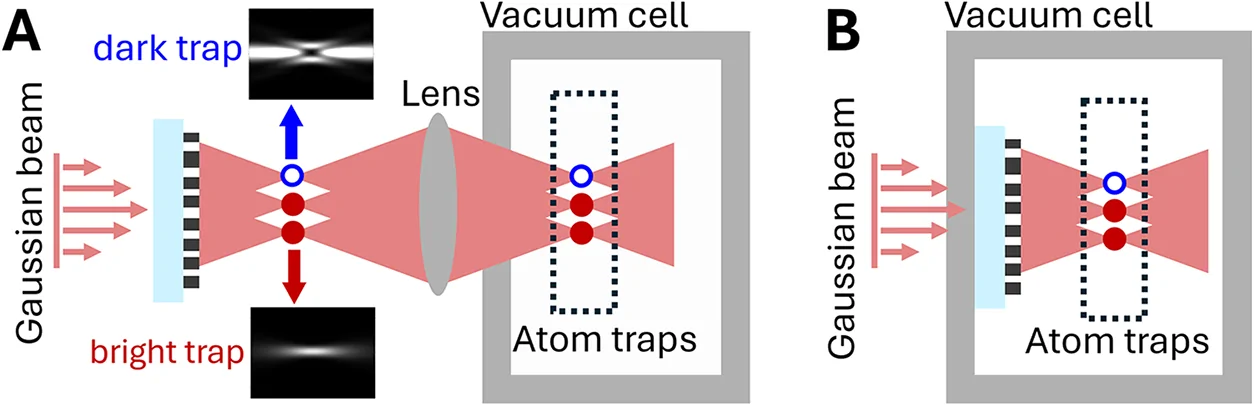
Two schemes for positioning a trap-forming metasurface with respect to the vacuum cell. (A) Atom-trapping setup with the metasurface placed outside of the vacuum cell. The dark traps (shown in blue) trap the atoms at the intensity minima when the laser frequency is blue-detuned from the atomic resonance, whereas bright traps (shown in red) trap the atoms at the intensity maxima when the laser is red-detuned. Relay optics reimage the intensity profiles into the vacuum cell. (B) Schematic of the atom trapping setup with the metasurface integrated inside the vacuum cell. The color convention is the same as (A).

Metasurfaces enable the direct generation of optical traps by applying subwavelength pixel-by-pixel phase control to the incident Gaussian beam. To guide the design of the phase profiles imparted by the metasurfaces, we must specify the target optical profiles at the focal plane. A bright trap is typically formed in the focal plane by tightly focusing a Gaussian beam to submicrometer size, whereas a dark trap can be created by overlapping two Gaussian beams with slightly different waists to interfere destructively, generating an intensity minimum at the center.^26^ Arrays of traps are then generated by repeating individual bright or dark traps. For applications in quantum information, the spacing between atoms is typically a few microns, to enable Rydberg interactions.^27^

The metasurface design problem is, then, to take an intensity distribution that forms arrays of bright and/or dark optical traps in the focal plane and identify the metasurface phase profile that will generate that desired distribution. This looks like a common metasurface design process that is often addressed using one of several versions of the Gerchberg-Saxton (G-S) algorithm; in particular, the G-S algorithm was previously used to design a TiO_2_ metasurface that generated an array of bright traps.^15,16^ A conventional G-S algorithm works well for arrays of bright traps because defining the target two-dimensional (2D) amplitude profile at the focal plane is generally sufficient to realize a three-dimensional (3D) tweezer.^15,16^

However, dark traps are more challenging to form because they require not only intensity minima in the focal plane but also high-intensity barriers on all sides of the trap in 3D space. Conventional G-S algorithms are not equipped to optimize for such a 3D intensity distribution. Instead of attempting to optimize for a 3D intensity distribution on multiple 2D planes, we instead optimized for the complex field (amplitude and phase) in the focal plane that can define both bright tweezers and bottle beams. Because a bottle beam can be formed by the interference of two Gaussian beams with different waist sizes, the desired amplitude and phase profile in the focal plane can be readily computed. The amplitude profile is the subtraction of two Gaussian profiles with different waists, and the phase profile is flat.

We thus use a modified G-S algorithm (illustrated in Figure 2(A)) to retrieve the optical phase profile of the metasurface ϕ­(*x*, *y*) that transforms a known input Gaussian beam with an amplitude profile *A*(*x*, *y*), into a target field *A*′(*x*, *y*)*e* ^*iϕ* ^′^(*x*,*y*)^ at the image plane. The algorithm starts with an initial random phase profile of the metasurface with symmetry along *x* = 0 and *y* = 0 to ensure the resulting optical fields have the same symmetry. The algorithm then proceeds through iterations, each consisting of steps 1–4:

1. **Forward propagation**: The complex field after passing through the metasurface, *A*(*x*, *y*)*e* ^*iϕ*(*x*,*y*)^ (green in Figure 2(A)), is numerically propagated to the image plane via the angular-spectrum method,^28^ resulting in the complex field *A*′(*x*, *y*)*e* ^*iϕ* ^′^(*x*,*y*)^ (magenta in Figure 2(A)).
2. **Applying image-plane constraints**: In the image plane (magenta in Figure 2(A)), we replace the propagated amplitude *A*′(*x*, *y*) in the region demarcated with a white box with a target amplitude. We replace the propagated phase ϕ′(*x*, *y*) across the entire computational domain. The enforcement of the phase constraint even outside of the region where we have traps ensures that traps near the edge of the array are not distorted.
3. **Backward propagation**: The newly constrained complex field from the image plane is then back-propagated to the metasurface plane via the angular-spectrum method, resulting in a new complex field *A*(*x*, *y*)*e* ^*iϕ*(*x*,*y*)^. The propagation is along –*z*.
4. **Applying metasurface-plane constraints**: In the metasurface plane (green in Figure 2(A)), the amplitude profile of the newly generated complex field through backward propagation, *A*(*x*, *y*), is replaced with the input Gaussian beam profile, while the phase ϕ­(*x*, *y*) is retained. This updated complex field then becomes the input for the next iteration.

**Figure 2:**
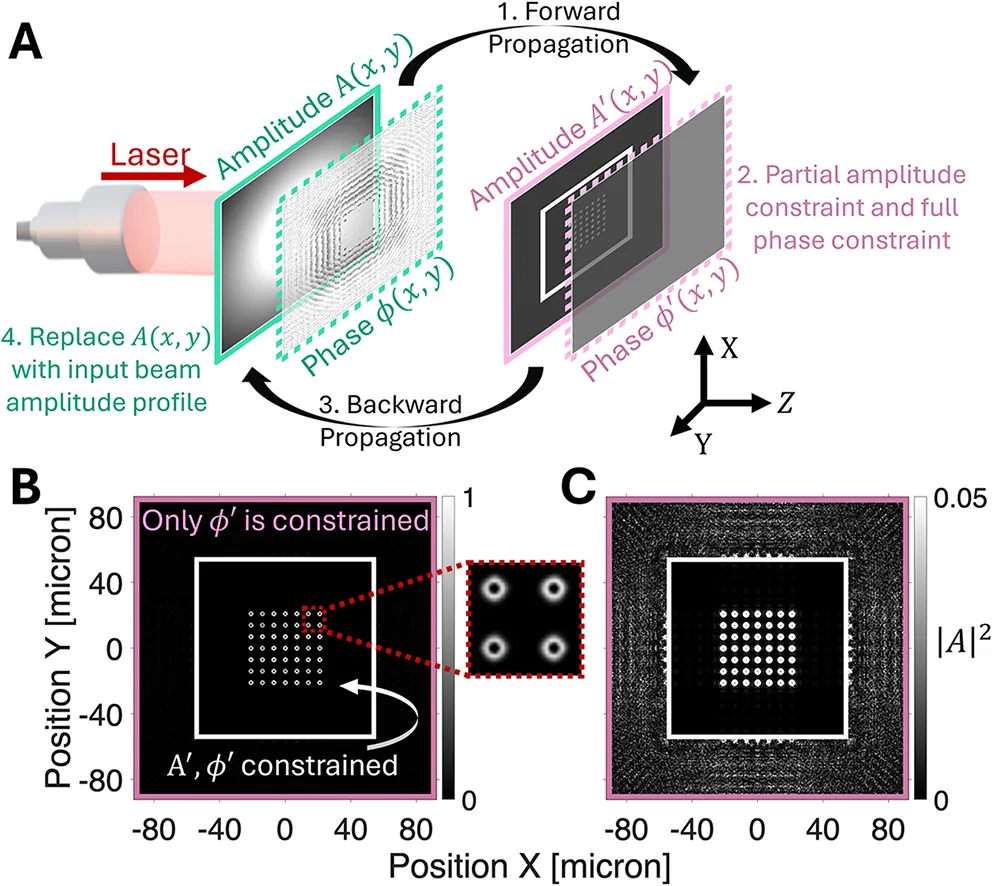
Modified Gerchberg-Saxton (G-S) algorithm for generating the bottle-beam array. (A) Flowchart of the modified G-S algorithm. Each iteration goes through four steps: (1) forward propagation from the metasurface to the image plane; (2) enforcement of both the target amplitude and phase profiles in the fully constrained domain, while only the phase profile is enforced in the partially constrained domain; (3) backward propagation from the image plane to the metasurface; (4) enforcement of the amplitude profile based on the input (usually Gaussian) beam. When the target amplitude *A*′(*x*, *y*) converges, the resulting metasurface phase ϕ­(*x*, *y*) can be implemented in hardware. (B) Simulated intensity profiles in the image plane forming an array of bottle-beam traps, generated using the algorithm in panel (A). Inset shows a magnified view of several bottle-beam traps. Panel (C) same profile as panel (B), but saturated to better show energy leaking into the partially constrained region.

This loop is repeated until *A*′(*x*, *y*) converges. The resulting phase profile on the metasurface ϕ­(*x*, *y*) can then be implemented via c-SOS meta-atoms. We demonstrate the results of this optimization process in Figure 2(B), where a 7 × 7 array of bottle-beam traps is formed, surrounded by darkness. To better see the contrast between the fully and partially constrained regions, we saturate the intensity scale in Figure 2(C), which shows that some light (unavoidably) leaks into the partially constrained region.

## Materials and Methods

We implemented our metasurfaces using crystalline silicon-on-sapphire (c-SOS) wafers, which offer several benefits over other materials that have been used for metasurfaces for atom-trap array generation: titanium dioxide (TiO_2_)^15,18^ and silicon nitride (SiN_*x*_).^16,19^ Though by now it is a well-studied material for metasurfaces, TiO_2_ remains less foundry-compatible because it is challenging to etch;^29^ fabrication typically relies on conformal atomic layer deposition through a lithographically defined mask followed by planarization.^30^ SiN_*x*_ is more foundry-compatible, but its lower refractive index is less favorable for meta-atom design and results in the need for higher-aspect-ratio structures.^31^

By contrast, crystalline silicon (c-Si) is the most mature material in semiconductor foundries and offers suitable optical properties in the near-infrared range. As shown in Figure S1, near the D_2_ resonance wavelengths of ^87^Rb (780 nm) and ^133^Cs (852 nm), c-Si has a refractive index of ∼3.6–3.7 and an extinction coefficient of less than 0.01. This enables the transmissive metasurface to imprint arbitrary phase profiles on the incident wave without significant optical absorption.

Our meta-atoms are cylindrical silicon pillars with a pitch of 360 nm, which is less than half the free-space wavelength to suppress higher diffraction orders and avoid spatial aliasing. The silicon thickness is 500 nm, a choice that balances practical fabrication constraints and the ability to achieve full 0 → 2π phase control. With pitch and thickness fixed, we vary the cylinder radius in 1 nm increments to generate a library of complex transmission coefficients, then select a radius range that provides a full 0 → 2π phase control while maintaining high transmittance. The corresponding discretized phase and amplitude responses of the meta-atoms are shown in Figure 3(A). The metasurface layouts are then generated by mapping the target phase from the modified G-S process (Figure 2) at each lattice site to the nearest available phase value, yielding the pillar radius at that site.

**Figure 3:**
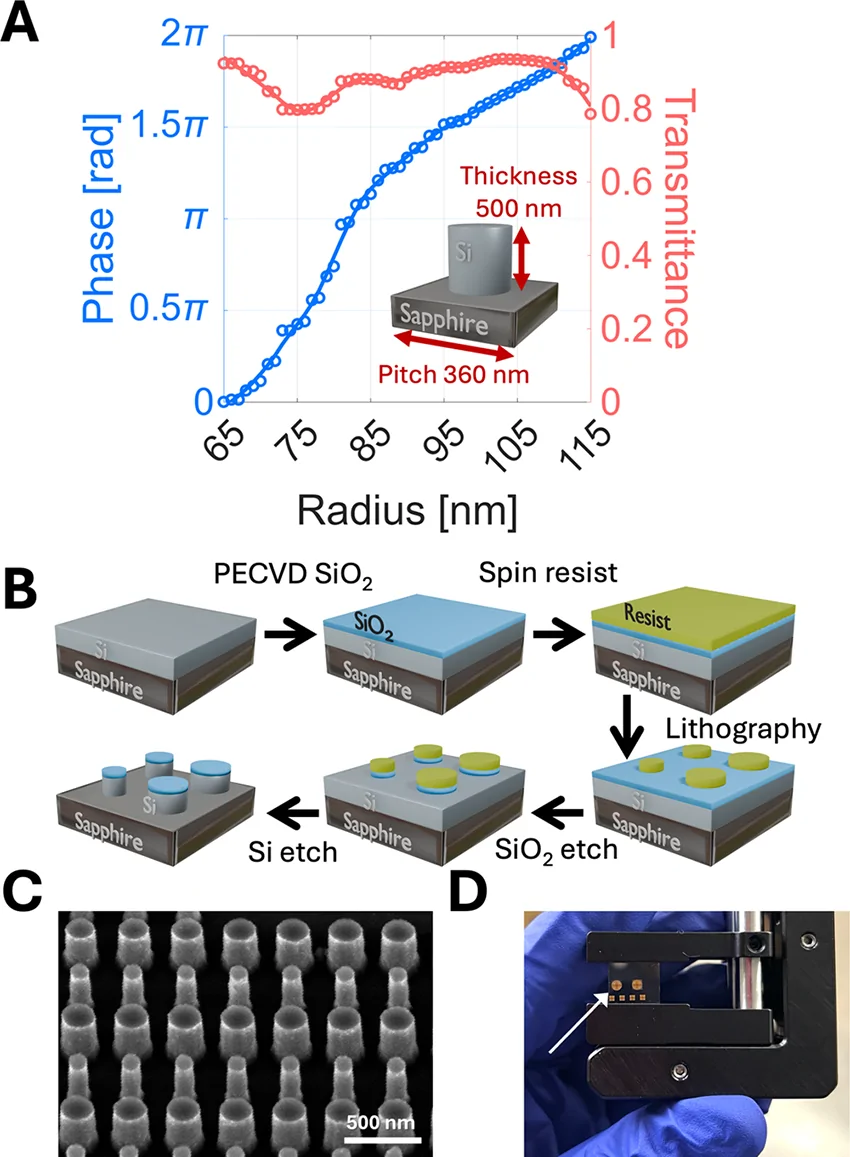
Realization of the designed phase profile using silicon-on-sapphire (c-SOS) metasurfaces. (A) Simulated transmittance and phase of a single unit cell as a function of the silicon cylinder radius. The height of the cylinder is 500 nm, with a period of 360 nm, on a sapphire substrate. The free-space wavelength of the incident beam in this simulation is 770 nm. The transmittance here assumes no reflection at the air-sapphire interface. (B) Fabrication processes of the silicon-on-sapphire metasurface. An SiO_2_ hard mask is first deposited on the silicon-on-sapphire substrate. The patterns are defined by electron-beam lithography and transferred to a hard mask via SiO_2_ etching. Then, silicon etching creates the silicon pillars, which remain covered with the SiO_2_ mask. Since the refractive index of the amorphous SiO_2_ is low, the residual SiO_2_ disk has a negligible influence on the performance of the metasurface. (C) SEM image of the metasurface on a tilted stage. (D) Photo of the fabricated metasurfaces mounted on a sample holder.

The fabrication flow is illustrated in Figure 3(B). First, SiO_2_ is deposited on the c-SOS wafer by plasma-enhanced chemical vapor deposition (PECVD). A positive e-beam resist (ZEP 520A) is spun, soft-baked, and exposed to write the patterns with varying radii that encode the designed phase profile. After development and a short O_2_ plasma descum, the pattern is transferred to the SiO_2_ by reactive ion etching (RIE) to create a hard mask. Next, the c-Si layer is etched by RIE to a target height of 500 nm using HBr/Cl_2_/O_2_. The O_2_ partial pressure is essential to achieve vertical side walls. The SiO_2_ hard mask is left on the c-Si pillars since it has a negligible impact on the optical performance. Scanning electron microscopy (SEM) at an angle of 45° was used to verify the fabrication, shown in Figure 3(C). A photograph of multiple finished c-SOS metasurfaces on one chip is provided in Figure 3(D). We note that the optical function of the metasurfaces is robust to reasonable fabrication errors, such as nonvertical side walls resulting from imperfect etching. As an example, we simulated the performance of the dark-trap array metasurfaces using hourglass-shaped pillars, as shown in Figure S5.

## Results

### Intensity Profile of Single Dark Trap

We first designed and fabricated a metasurface for generation of a single dark trap (Figure 4), similar to one of the designs using accelerating beams.^32^ The specific target dark trap consists of the interference of two Gaussian beams with waists *w*_1_ = 1.33 μm and *w*_2_ = 0.7 μm, such that there is destructive interference at the beam waist. As a result, the target amplitude profile is the subtraction of these two Gaussian profiles at the beam waists, and the phase profile is flat.

**Figure 4:**
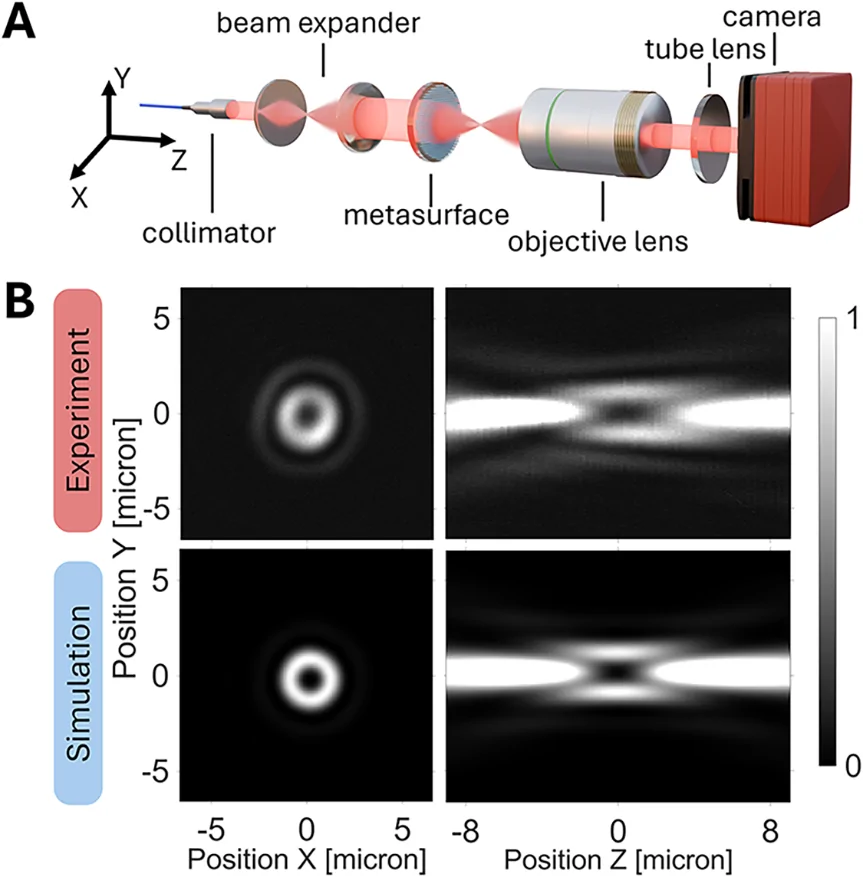
Generation and measurement of a single dark trap. (A) Experimental setup for imaging the intensity profile after the metasurface. A Gaussian beam at 780 nm is launched from a single-mode fiber with a collimator and expanded to the desired beam waist. The metasurface is mounted on a motorized *XYZ* stage combined with a tip-tilt stage. The intensity profiles of the optical traps are acquired with a microscope while scanning the metasurface along the *Z*-direction. (B) Experimental (top row) and simulated (bottom row) intensity profiles. Left plots show the *XY* intensity profiles in the focal plane at *Z* = 350 μm, and right plots show the *YZ* intensity profiles at *X* = 0 μm. All profiles are normalized to the maximum intensity of the corresponding *XY* intensity profile.

The optical characterization setup is shown in Figure 4(A). A 780 nm Gaussian beam from a single-mode fiber was expanded to the designed input waist *w*_0_ = 0.13 mm before illuminating the metasurface. The metasurface was placed at the beam waist of the input Gaussian beam and mounted on motorized *XYZ* and tip-tilt stages. These stages were carefully adjusted to ensure that the metasurface center was aligned with the Gaussian beam center and the beam was strictly perpendicular to the metasurface. The dark trap formed at a working distance of 0.35 mm. To measure the intensity profile, a microscope was built on the other side of the trap with an objective lens (*f*_1_ = 4.5 mm, NA = 0.65), a tube lens (*f*_2_ = 50 mm), and a monochrome camera (Allied Vision Alvium 1800 U-501m NIR). The metasurface is translated along the optical axis (*Z*) to acquire a full 3D map of the intensity. Strictly speaking, we should translate the microscope to keep the input beam unchanged on the metasurface; however, because the beam’s Rayleigh length is much longer than the scanning range, we instead translate the metasurface, which is simpler than moving the entire microscope.

Figure 4(B) compares measured and simulated intensity profiles, which are both normalized to the peak intensity in the focal plane. The measured *XY* profile shows the expected “donut” shape in the focal plane, with a low-intensity center. The measured *YZ* profile at *X* = 0 shows a bottle-beam geometry. Because of the camera’s limited dynamic range, we do not report a reliable trap depth value here. Nonetheless, the close agreement between the experiment and simulation results confirms that the designed and fabricated metasurface generates the dark trap as intended.

### Intensity Profiles for One- and Two-Species Arrays

We further demonstrate metasurface-generated optical trap arrays in both single-species and dual-species configurations. The target amplitude profile of an array is generated by placing copies of the single-trap target amplitude profile at the desired lattice sites. We designed arrays for single-species traps (all bright and all dark) or a mixture of bright and dark traps. The target phase profile is set to be flat across the entire trapping plane to generate the dark traps and improve the uniformity across the array. In Figure 5, we compare the experimentally measured and simulated intensity distributions for (A) a dark-trap array, (B) a bright-trap array, and (C) an interleaved dual-species array that combines both types of traps using the same optical setup as in Figure 4(A).

**Figure 5:**
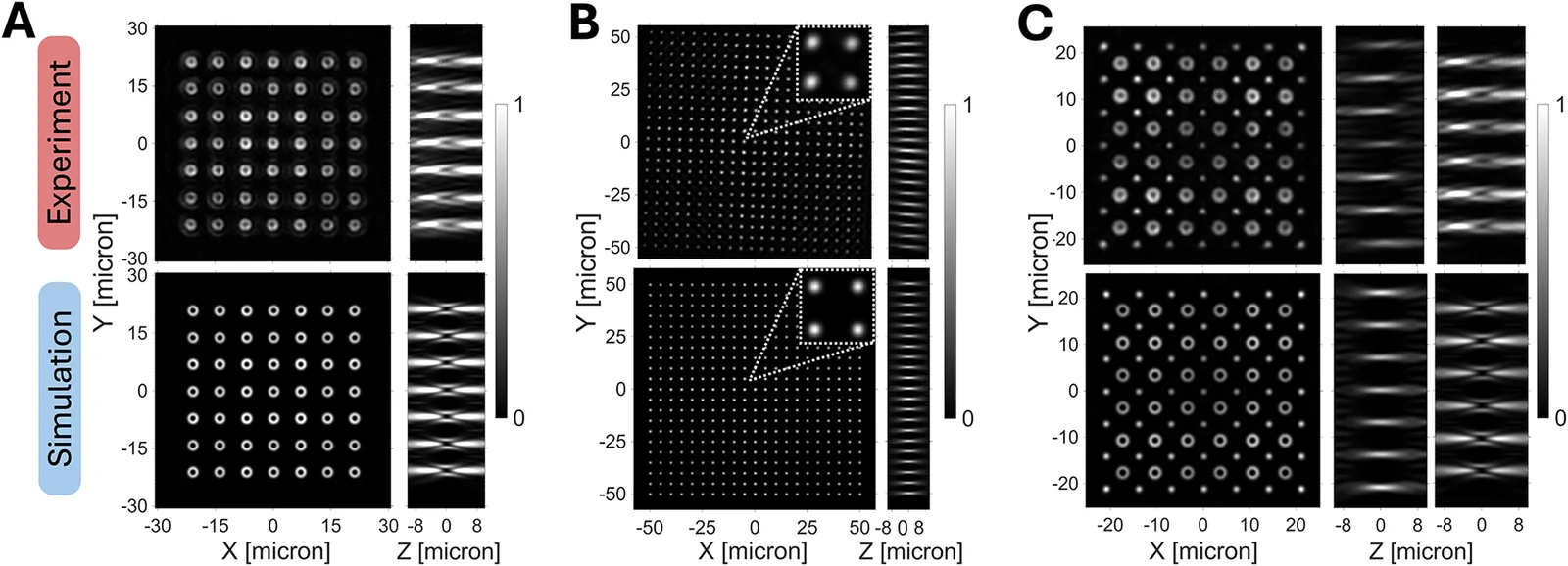
Experimental and simulated intensity distributions for dark, bright, and dual-species trap arrays. Experiments (top row) and simulations (bottom row). The left plots of each panel show the *XY* intensity profiles in the focal plane, at *Z* = 350 μm. The right plots of each panel show the *YZ* intensity profiles across trap sites. In panels (A) and (B), the profiles are along *X* = 0 μm; for panel (C), the profiles are along *X* = 0 μm (bright traps) and *X* = 3.5 μm (dark traps). All profiles are normalized to the maximum intensity of the corresponding *XY* intensity profile, with line profiles shown in Figure S6. (A) 7 × 7 dark-trap array. (B) 21 × 21 bright-trap array. The insets in the *XY* plots show magnified views of the four traps at the center. (C) 7 × 7 bright-trap array interleaved with 6 × 6 dark-trap array.

For the single-species arrays shown in Figure 5(A,B), the metasurfaces generate dark (blue-detuned) and bright (red-detuned) traps, respectively. We selected modest array sizes and lattice periods to make it easy to image the arrays, but the approach shown here can be used for much larger arrays with spacing down to about 3 μm (or even smaller, with higher NA), though the bright traps can be somewhat closer together than the dark traps.

The dark-trap array in Figure 5(A) consists of a 7 × 7 periodic lattice with a period of 7 μm, designed using the same parameters as the single dark trap in Figure 4(B). The bright-trap array in Figure 5(B) consists of 21 × 21 traps with a period of 5 μm; each bright trap has a Gaussian profile with a waist of 0.94 μm, which matches the trap size of dark traps. The measured *XY* intensity profiles reveal periodic arrays of donut-shaped dark traps in Figure 5(A) and bright focal spots in Figure 5(B), while the *YZ* cross sections at *X* = 0 confirm strong axial confinement in both cases. Excellent agreement was obtained between the simulations and the measured intensity profiles.

We also designed a metasurface capable of simultaneously generating interleaved bright and dark traps (Figure 5(C)), an arrangement of interest for two-species architectures for quantum error correction.^33,34^ In this configuration, the trapping laser wavelength would be chosen to be between the resonance wavelengths of the two atomic species, making it red-detuned for one species and blue-detuned for the other. The focal plane of the metasurface in Figure 5(C) contains 7 × 7 bright traps interleaved with a 6 × 6 dark-trap array, forming a checkerboard pattern, as shown in the *XY* intensity profile. The *YZ* cross section at *X* = 0 μm displays the bright-trap array, while the cross section at *X* = 3.5 μm corresponds to the dark-trap array.

## Discussion

The results in Figure 5 show that the metasurfaces can generate trap arrays for one or two atomic species, using a single laser and with no additional optical components. To quantitatively evaluate the performance of these metasurface-generated trap arrays, we calculated the trap depth at each individual trapping site and the standard deviation across the entire array for dark- and bright-trap arrays. In this analysis, we assume the input trapping laser is a Gaussian beam with a waist of roughly 130 μm at a power of 1 W incident on the metasurface. While the laser wavelength varies depending on the specific application settings, such as the atomic species or the availability of a high-power laser, we use λ = 780 nm as an example for the following calculations, since the same metasurface design performs well at nearby wavelengths (see, for example, Figure S4).

We use the trap depth at a given input power as our figure of merit, rather than the efficiency of light delivery to the region of the traps. A higher efficiency does not necessarily translate to better dark traps since deformation of the dark traps can cause leakage of atoms. For the dark traps, the trap intensity depth is defined as the intensity difference between the minimum at the trap center and the nearby “escape point”, as illustrated in Figure 6(A,B). The escape point is the location where an atom would be the most likely to exit the trap. To identify this point, we use a modified Dijkstra’s algorithm^35^ on the three-dimensional intensity distribution to search for the escape path that minimizes the maximum potential barrier along the route. The escape point is then defined as the maximum intensity along this optimal path. We note that the escape points in the dark traps are not located in the *X*–*Y* plane; to visualize the exact location, in Figure 6(B), we plot the cross-section intensity profile across the trap, taking the cut that crosses the center point and the calculated escape point. Note that the central traps have several equivalent escape points due to symmetry, and our algorithm finds one of them. Using this definition, we calculate the trap depths for every site across the 7 × 7 dark-trap array, resulting in the trap-depth distribution shown in Figure 6(C). The average trap depth is 55 kW/cm^2^ with a coefficient of variation (CV) of 0.22, where CV is defined as the standard deviation divided by the mean.

For the bright traps, we define the trap intensity depth as the difference between the peak intensity at the trap center and the surrounding background intensity, as shown in Figure 6(D,E). Because the background intensity is negligible compared to the peak intensity, the trap depth can be just treated as the peak intensity. Using this definition, we calculate the trap depths for every site across the 21 × 21 bright-trap array, giving us the trap-depth distribution shown in Figure 6(F). The average trap depth is 65 kW/cm^2^ with CV = 0.12.

We found that it is significantly more energy efficient to form bright traps than dark traps. For a given amount of laser power and designing a bright-trap array and a dark-trap array with similar intensity trap depths, the bright-trap array would have approximately nine times more traps than the dark-trap array. This increased power consumption for dark traps is because the optical field must form a bottlelike distribution that surrounds the trap center, rather than being focused on the trap center as in bright traps. This bottlelike distribution takes a larger volume and thus leads to a lower intensity contrast. While bright traps are more power-efficient, dark traps may still be preferred for applications requiring long coherence time and minimal scattering rate.^27,36^ We also investigated the sensitivity of the metasurfaces to input beam misalignment for the dark-trap arrays. Our simulations show that the metasurfaces are robust to moderate misalignment in the incident angle (Figure S2) and the beam center (Figure S3).

In summary, we experimentally demonstrated a CMOS-compatible silicon-based metasurface platform for atom trapping applications using near-infrared lasers. We realized metasurfaces that generate arrays of bright traps, dark traps, and interleaved dark and bright arrays using a single input laser. Compared to approaches based on active beam-shaping devices (e.g., AODs), our metasurfaces are passive, eliminating noise due to active components and potentially improving trap stability. The metasurfaces and the resulting trap arrays can be scaled to nearly arbitrary sizes (e.g., see Figures S7 and S8), limited primarily by fabrication time if using serial techniques such as electron-beam lithography, and the availability of high-power lasers. The metasurfaces are also highly compact and thus can be integrated inside the vacuum cell, supporting miniaturized quantum setups. This paper positions crystalline silicon-based metasurfaces as a scalable tool for atom-based quantum technologies. Looking ahead, we envision metasurfaces as an element of a hybrid architecture for neutral-atom platforms. In such an architecture, a passive metasurface provides a background trapping array with complex trapping profiles and/or a large number of trapping sites, while active optical components (e.g., AODs) offer dynamic functionalities such as atom rearrangement.

**Figure 6:**
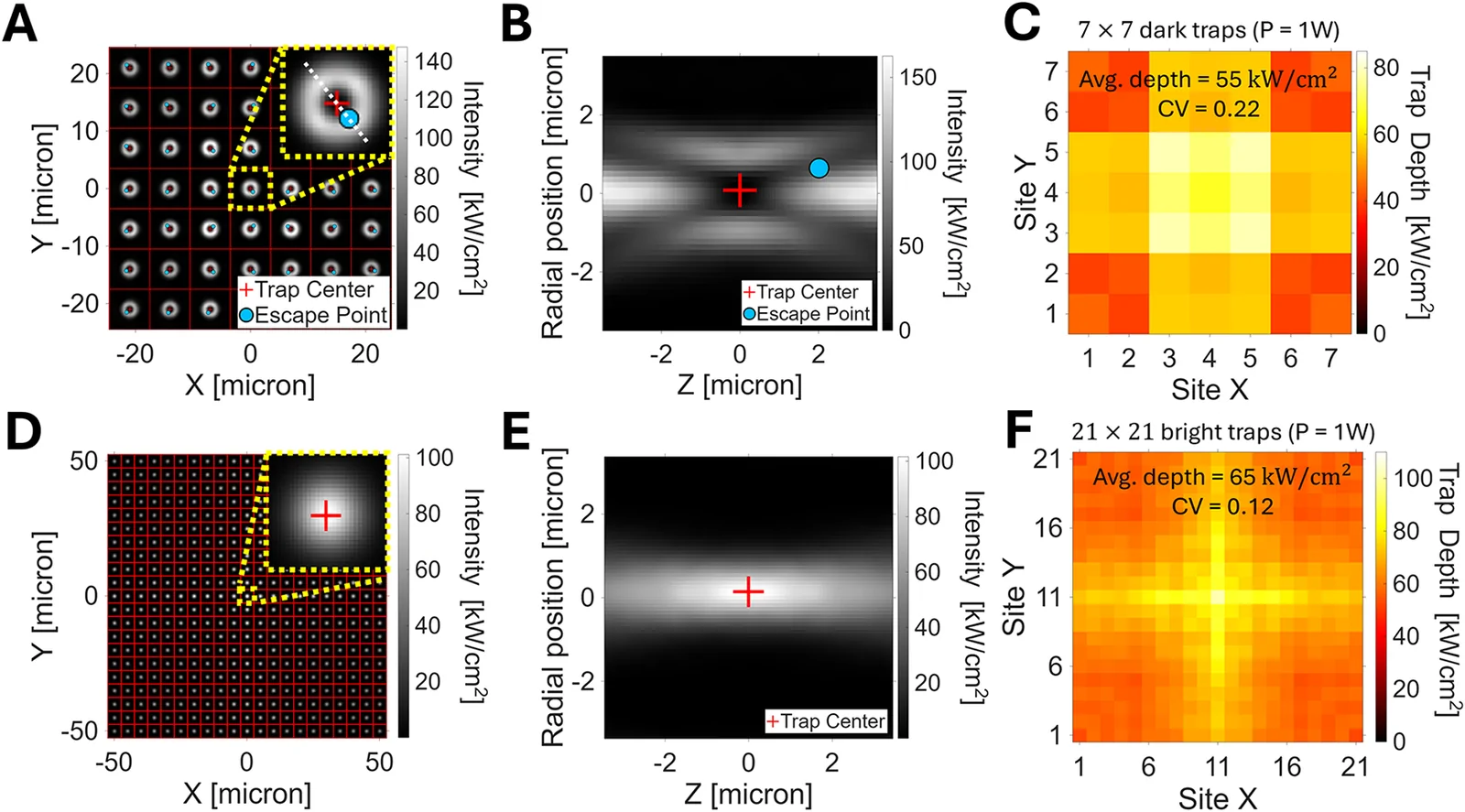
Trap depth analysis of dark (A–C) and bright (D–F) trap arrays. (A) XY intensity profile for a 7 × 7 dark-trap array. The inset shows a magnified view of a single dark trap, where the red cross marks the trap center (intensity minimum) and the blue circle indicates the calculated escape point. Note that in the central trap sites, there are multiple escape points due to symmetry, and we plot the one found by our algorithm. (B) “Radial” cross-section across a single dark trap showing 3D confinement, where the specific cut is along the direction given by the trap center and the projection of the escape point on the *X*–*Y* plane, as shown in the inset of panel (A). (C) Trap-depth distribution heatmap for the 7 × 7 dark array at 1 W input. (D) XY intensity profile for a 21 × 21 bright-trap array. The inset shows a magnified view of the central bright trap, where the red cross marks the trap center (intensity maximum). (E) Radial cross-section of the central bright trap, showing 3D confinement. (F) Trap depth distribution heatmap for the 21 × 21 bright array at 1 W input.

## Supplementary Material



## Notes

Optical properties of crystalline silicon on sapphire; effects of an off-angle incident Gaussian beam; effects of an off-center incident Gaussian beam; performance of the metasurface for dark-trap-array generation at a different input wavelength; performance of the metasurface for dark-trap-array generation considering fabrication errors; intensity line cuts through the experimental intensity profiles; uniformity-optimized bright-trap arrays; design of a large array of dark traps; and a comparison between trapping methods (PDF)

## ∇

QuEra Computing, Inc., 1380 Soldiers Field Road, Boston, Massachusetts 02135, USA
