# Supplementary material for: Silicon-On-Sapphire Metasurfaces Generate Arrays of Dark and Bright Traps for Neutral Atoms
Source: ACS Photonics. 2026 Jul 6;13(16):4870–7. doi: 10.1021/acsphotonics.6c01311 (PMC13495955; doi:10.1021/acsphotonics.6c01311)
Supplement: Supplementary file 1 [file ph6c01311_si_001.pdf]

Supporting Information for: Silicon-on-sapphire metasurfaces generate arrays of dark and bright traps for neutral atoms

Chengyu Fang,[†] Minjeong Kim,[†] Hongyan Mei,[†] Xuting Yang,[‡] Zhaoning Yu,[†] Yuzhe Xiao,[†,¶] Sanket Deshpande,[†] Preston Huft,[§,#] Alan M. Dibos,[‖] David A. Czaplewski,[‖] Mark Saffman,[§,⊥] Jennifer T. Choy,[†,‡,§] and Mikhail A. Kats[*,†,‡,§]

†*Department of Electrical and Computer Engineering, University of Wisconsin-Madison, 1415 Engineering Drive, Madison, WI 53706, USA*

‡*Department of Materials Science and Engineering, University of Wisconsin-Madison, 1509 University Avenue, Madison, WI 53706, USA*

¶*Department of Physics, University of North Texas, 210 Avenue A, Denton, TX 76201, USA*

§*Department of Physics, University of Wisconsin-Madison, 1150 University Avenue, Madison, WI 53706, USA*

‖*Center for Nanoscale Materials, Argonne National Laboratory, Lemont, IL 60439, USA*

⊥*Infleqtion, Madison, WI, 53703, USA*

#*Present address: QuEra Computing, Inc., 1380 Soldiers Field Road, Boston, MA 02135, USA*

E-mail: *mkats@wisc.edu

Supplementary Materials

1. Optical properties of crystalline silicon on a sapphire substrate

2. Effects of an off-angle incident Gaussian beam

3. Effects of an off-center incident Gaussian beam

4. Performance of the dark-trap-array metasurface at a different input wavelength

5. Performance of the dark-trap-array metasurface considering fabrication errors

6. Intensity line cuts through the experimental intensity profiles

7. Uniformity-optimized bright-trap arrays

8. Designing a large array of dark traps

1. Optical properties of crystalline silicon on a sapphire substrate

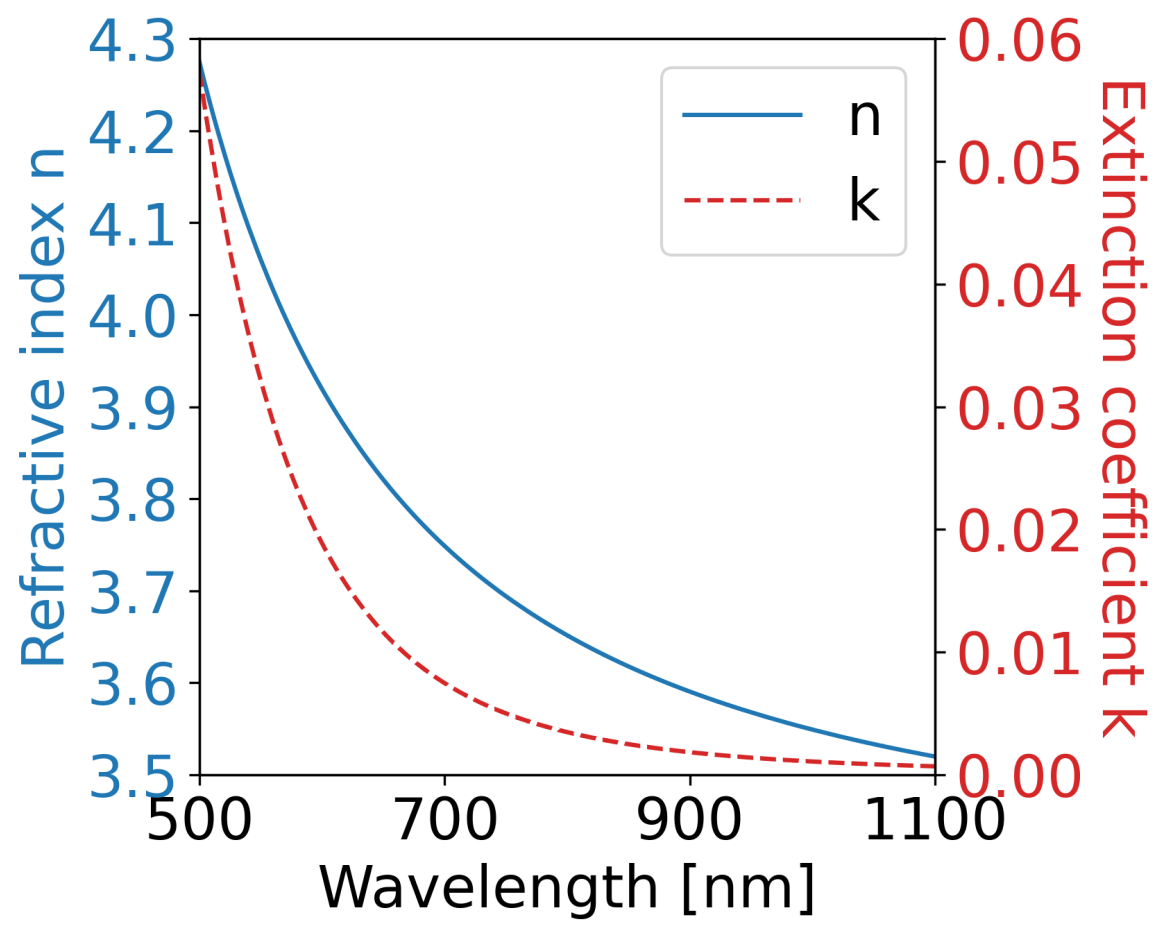

Figure S1: Wavelength-dependent complex refractive index $n + ik$, measured using spectroscopic ellipsometry. The real part n (blue solid, left y-axis) is relatively high ($\sim$3.5–4.3) from 500 to 1100 nm, while the extinction coefficient k (red dashed, right y-axis) remains low (< 0.01 for wavelengths longer than $\sim$700 nm).

2. Effects of an off-angle incident Gaussian beam

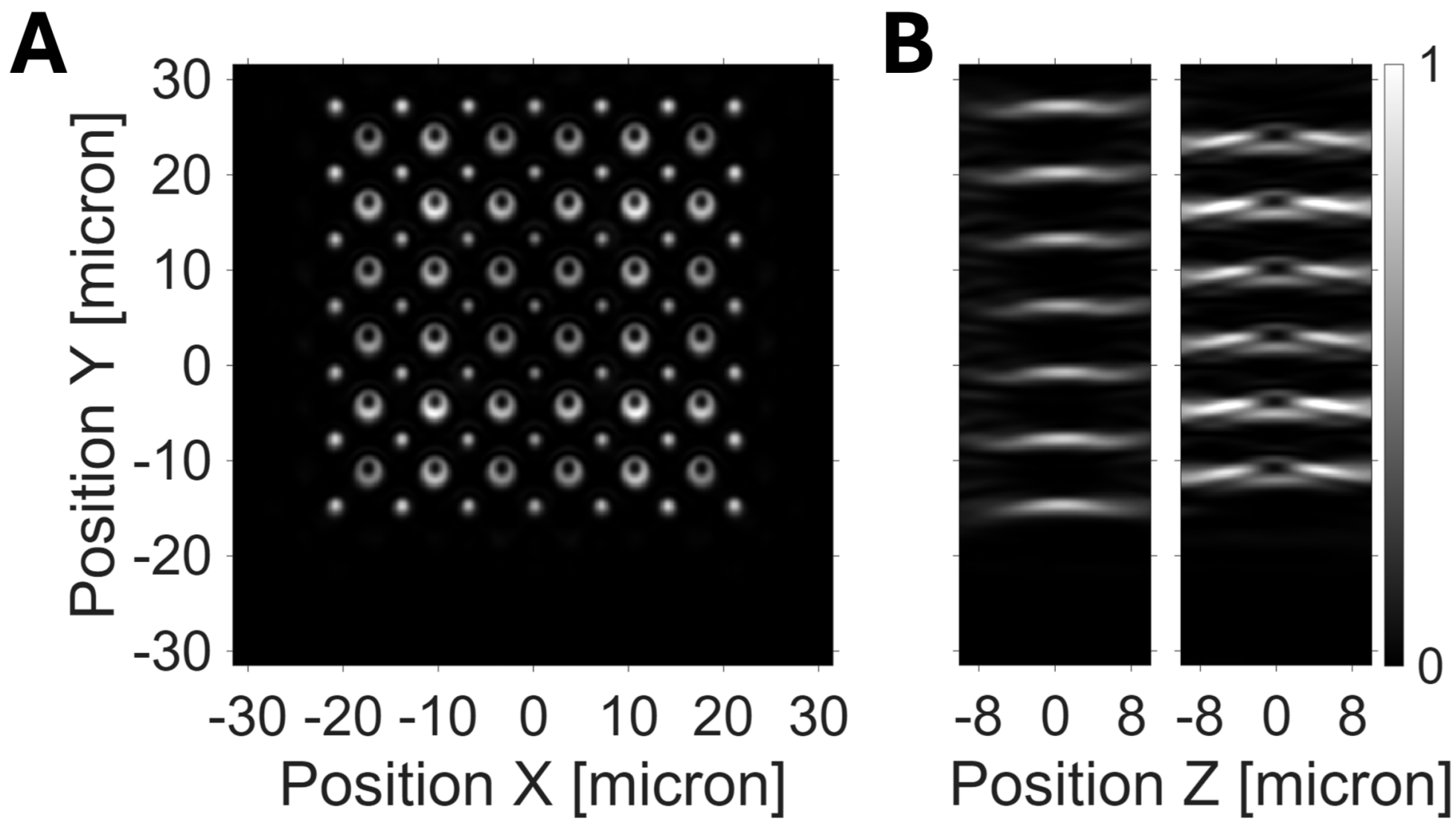

Figure S2: Simulated intensity profile of a two-species trap array generated by a metasurface, assuming the incident beam is 1° off the normal angle. The metasurface design is the same as the one in Fig. 5(C). **(A)** XY intensity profile in the focal plane at $Z = 350$ μm. **(B)** YZ intensity profiles along $X = 0$ μm (bright traps) and $X = 3.5$ μm (dark traps).

3. Effects of an off-center incident Gaussian beam

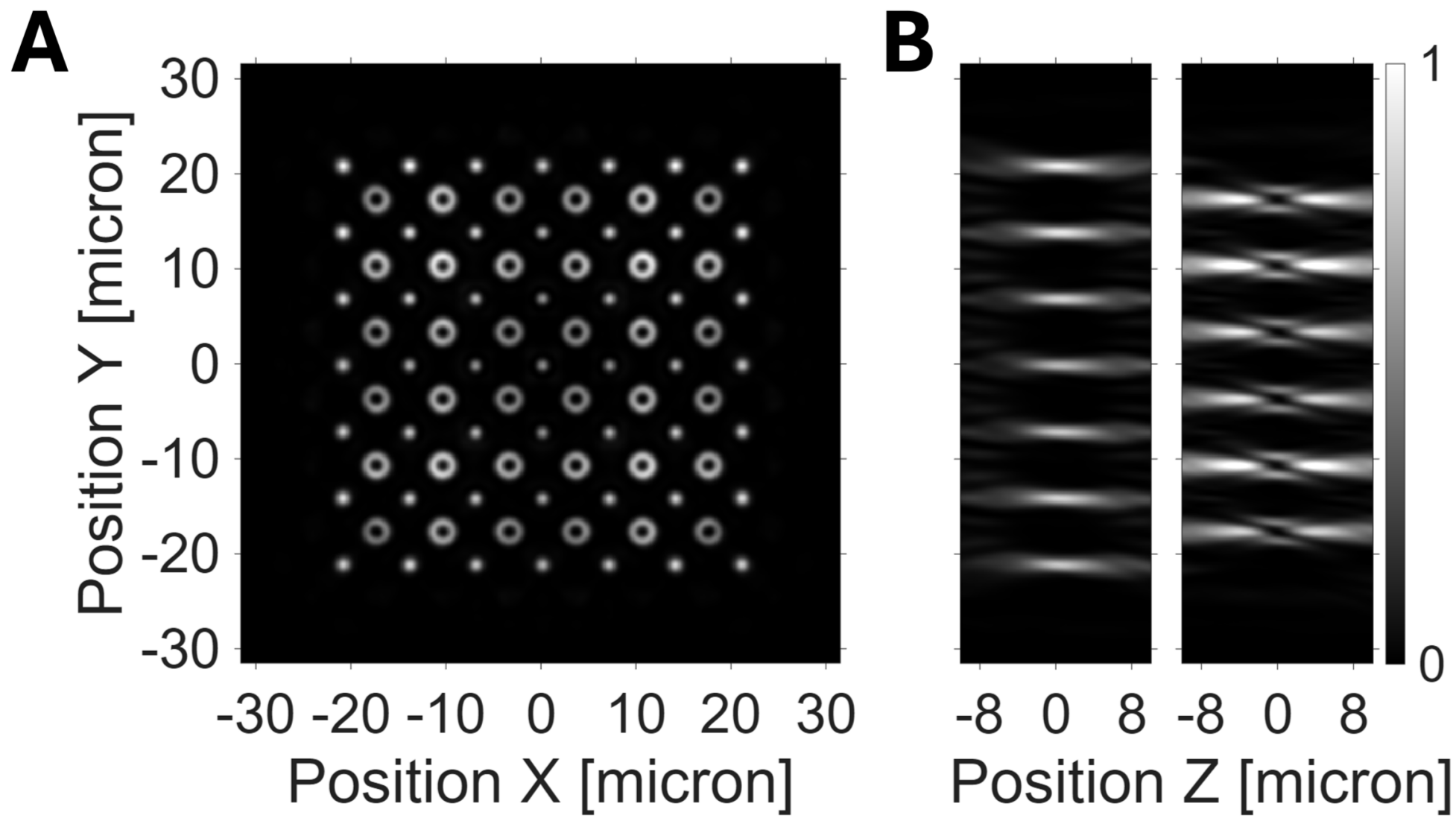

Figure S3: Simulated intensity profile of a two-species trap array generated by a metasurface, assuming the incident beam is 20 μm off the center. The metasurface design is the same as the one in Fig. 5(C). **(A)** XY intensity profile in the focal plane at $Z = 350$ μm. **(B)** YZ intensity profiles along $X = 0$ μm (bright traps) and $X = 3.5$ μm (dark traps).

4. Performance of the dark-trap-array metasurface at a different input wavelength

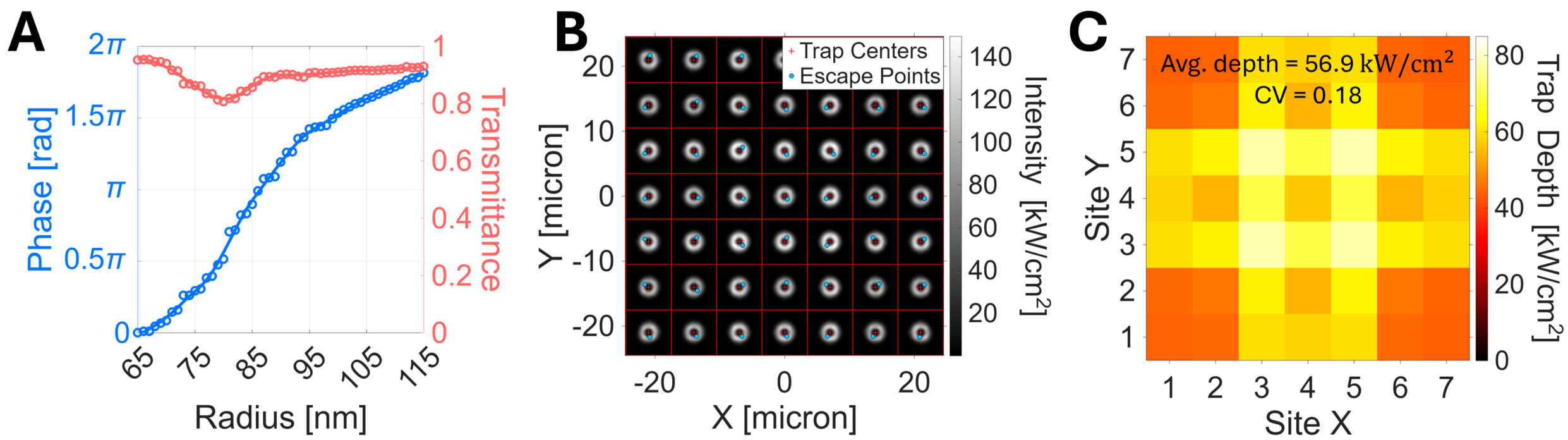

Figure S4: Performance of the dark-trap metasurface designed for an input wavelength of $\lambda = 770$ nm but operating at $\lambda = 800$ nm. **(A)** Simulated transmittance and phase of a single unit cell as a function of the silicon cylinder radius. The height of the cylinders is 500 nm, with a period of 360 nm, on a sapphire substrate. The free-space wavelength of the incident beam in this simulation is 800 nm. **(B)** Simulated XY intensity profile of the 7×7 dark-trap array in the focal plane. **(C)** Trap depth distribution heatmap for the array at 1 W input power.

5. Performance of the dark-trap-array metasurface considering fabrication errors

In the processes of metasurface fabrication, the shape of the silicon pillar might differ from the ideal cylindrical shape. As an example, due to imperfect etching processes, the sidewall of the pillars may not be vertical. Here, we simulated the performance of the dark-trap array metasurface with hourglass-shaped sidewalls, as shown in Fig. S5. We observe that the metasurface still generates a dark-trap array but with a slightly lower average trapping depth and similar CV.

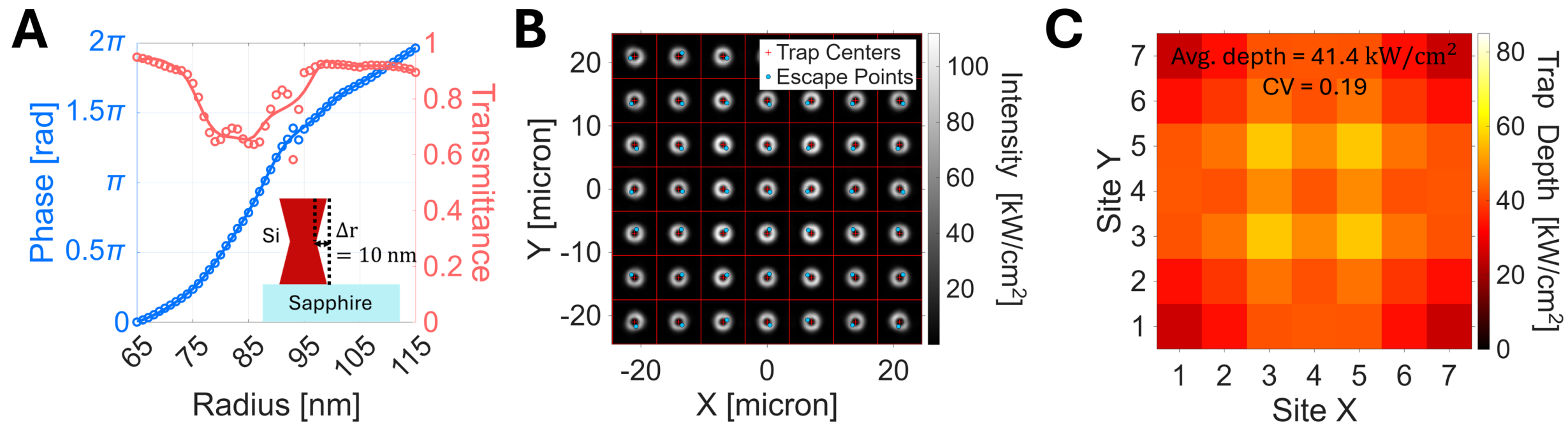

Figure S5: **(A)** Simulated transmittance and phase response of a single unit cell as a function of the silicon pillar radius. The pillars are hourglass-shaped to represent fabrication imperfections, with a radius waist at the middle that is 10 nm smaller than the pillar radius. **(B)** Simulated XY intensity profile of the 7×7 dark-trap array in the focal plane. **(C)** Trap depth distribution heatmap for the array at 1 W input power.

6. Intensity line cuts through the experimental intensity profiles

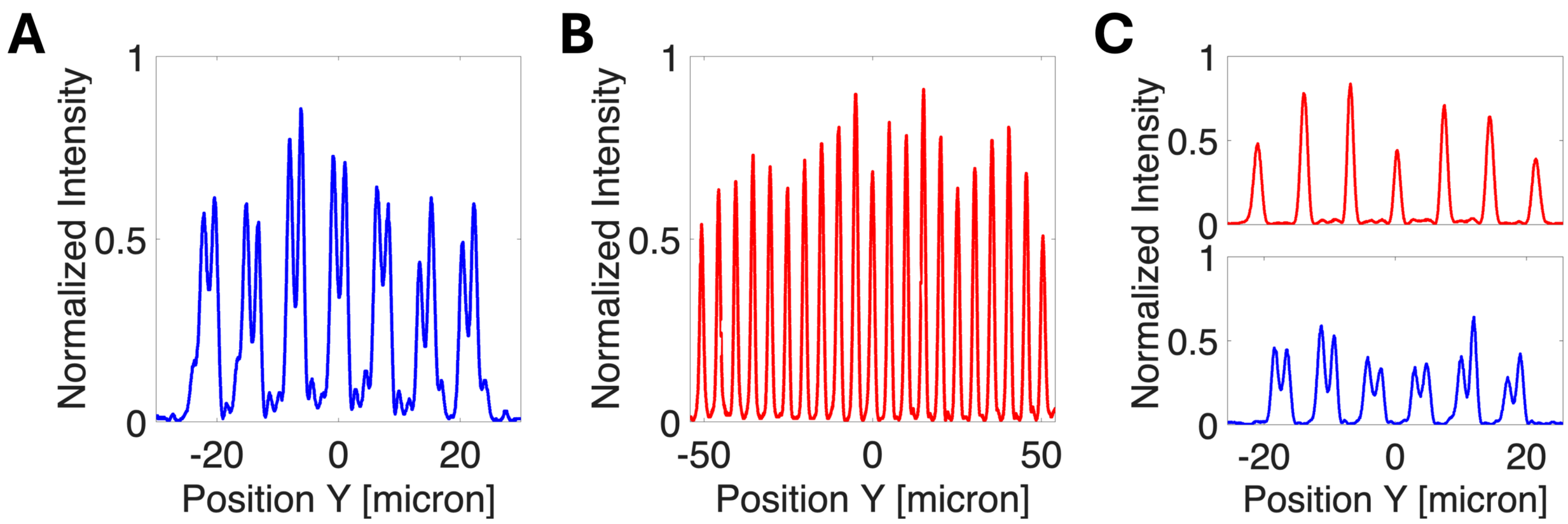

Figure S6: Intensity line profiles that cut through the experimental plots in Fig. 5(A-C) in the main text. The intensity is normalized to the highest intensity across the entire focal plane. **(A)** Intensity of the dark-trap array in the focal plane at $x = 0$ μm. **(B)** Intensity of the bright-trap array in the focal plane for $x = 0$ μm. **(C)** Intensity of the two-species trap array at focal plane for $x = 0$ μm (bright traps) and $x = 3.5$ μm (dark traps).

7. Uniformity-optimized bright-trap arrays

Though this was not our singular figure of merit in Fig. 5(B) of the main text, metasurfaces are capable of generating large bright-trap arrays with very high uniformity. For Fig. S7, we optimized an array with 25195 bright traps, with average trap depth of 41.81 $\mathrm{kW/cm^2}$ and CV=1.27%, assuming an input power of 20 W. In this simulation, we used the weighted G-S algorithm without any phase constraints to maximize the trap uniformity. The uniformity reported here is significantly better than that of the metasurfaces designed with phase constraints in the main text. This is because, without phase constraints, the G-S algorithm has additional degrees of freedom available to optimize the trap-depth uniformity at the focal plane.

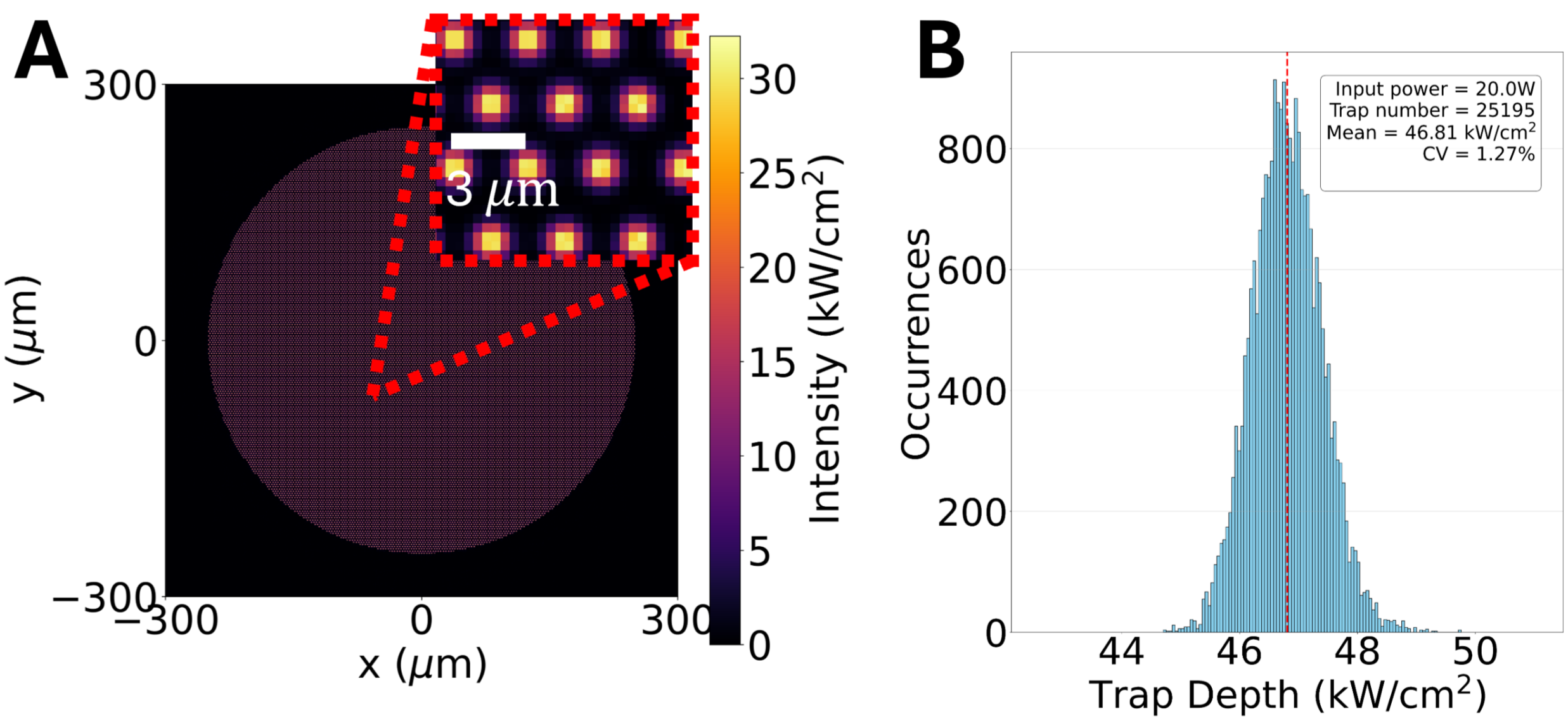

Figure S7: **(A)** Simulated intensity profile at the focal plane for a hexagonally arranged circular bright-trap array with pitch of 3 μm and radius of 250 μm. This array was optimized for trap uniformity. The inset shows a zoomed-in view of bright traps with beam waist of 0.9 μm. **(B)** Histogram of intensity trap depth across the entire array (25195 trap sites).

8. Designing a large array of dark traps

In this section, we demonstrate that metasurfaces are capable of generating large dark-trap arrays with moderate uniformity. While the uniformity can be further optimized, it is challenging to make dark-trap arrays with similar uniformity to bright-trap arrays (like in Fig. S7) because dark traps are more geometrically complex than bright traps, and therefore require more degrees of freedom to form, leaving fewer degrees of freedom to optimize uniformity. In Fig. S8, we demonstrated 3259 dark traps with average trap depth of 38.49 kW/cm^2, assuming an input power of 20 W. The resulting CV = 11.49%. We further characterize the variation in trap frequency and trap-center position across the array. The three trap frequencies at each site (Fig. S8(C)) are computed from the Hessian matrix of the local trapping potential, whose eigenvectors define the principal modes of the trap. The trap-center position, measured relative to its ideal position in the array, have standard deviations of 0.127 μm, 0.14 μm, and 0.069 μm along x, y, and z, which are small compared to the array lattice constant.

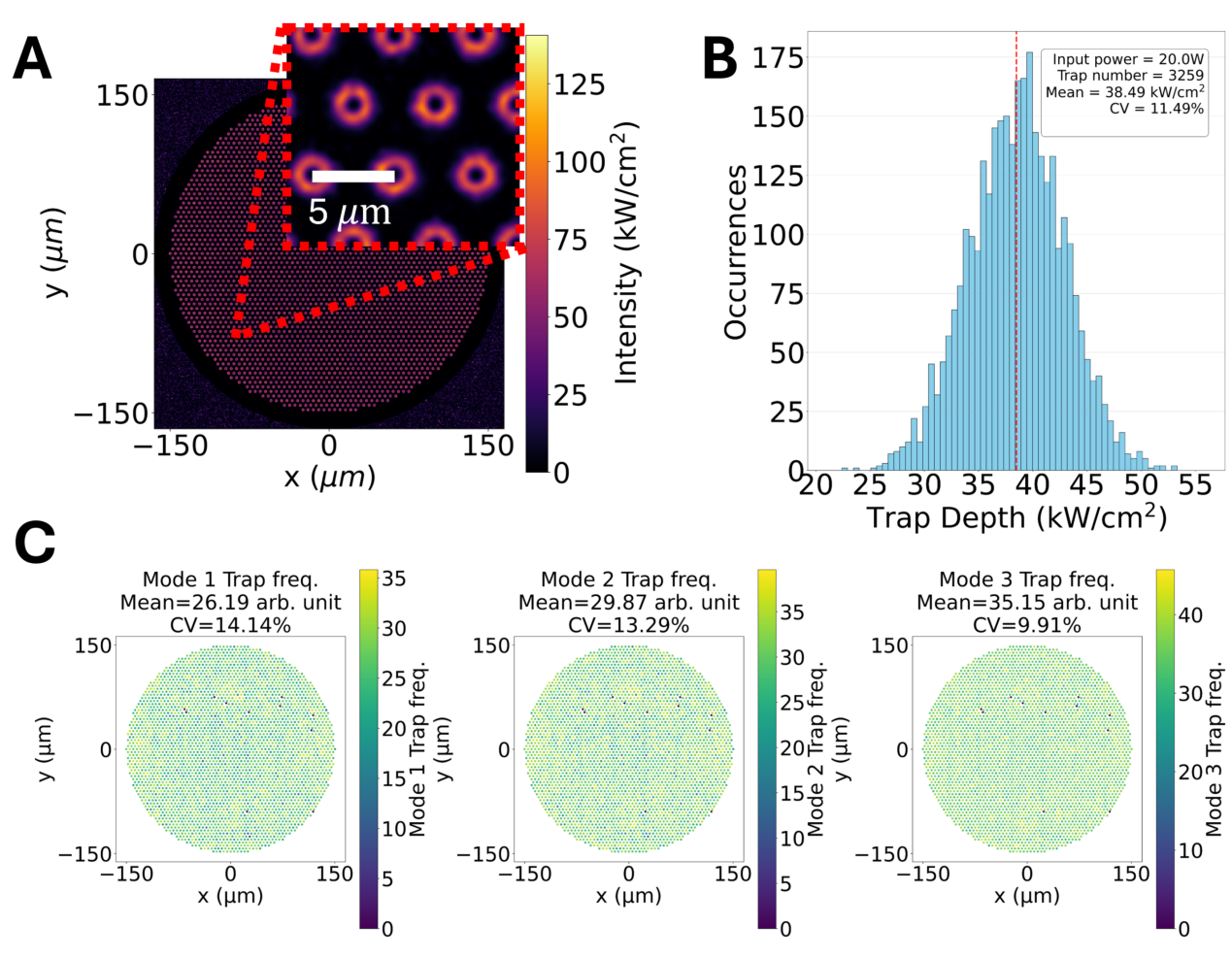

Figure S8: **(A)** Simulated intensity profile at the focal plane for a hexagonally arranged circular dark-trap array with pitch of 5 μm and radius of 150 μm. The inset shows a zoomed-in view of representative dark traps, which can be described as destructive interference between two Gaussian beams with waists $w_1 = 1.33$ μm and $w_2 = 0.7$ μm. **(B)** Histogram of the intensity trap-depth metric across the entire array (3259 trap sites). **(C)** Maps of the calculated trap frequencies for the three principal motional modes at each trap site across the array.

9. Comparison between trapping methods

Table S1 summarizes the optical devices that have been used to generate atom-trap tweezer arrays, including active devices (spatial light modulators, digital micromirror devices, acousto-optic deflectors) and passive devices (amplitude masks, microlens arrays, metasurfaces). For each device, we list the reframe rate, the largest experimentally demonstrated number of optical traps, the main challenges that each method is facing, and the largest number of optical tweezers that have been experimentally demonstrated.

Table S1: Comparison of optical devices used to generate atom-trap tweezer arrays.

Device	Refresh rate	Challenges	Max. optical tweezers demonstrated
Spatial light modulator	sub kHz	Limited pixel count; diffraction efficiency	∼12,000 sites[1]
Digital micromirror device	∼kHz	Limited pixel count; power efficiency	20 sites[2]
Acousto-optic deflector	∼MHz	Limited radio frequency tones	512 sites[3]
Amplitude mask	static	Power efficiency; static	1,225 sites[4]
Microlens array	static	Filling-factor loss; static	∼3,000 sites[5]
Metasurface	static	Static	360,000 sites[6]

[1]Manetsch et al. *Nature* **2025**, *647*, 60–67.
[2]Stuart and Kuhn. *New J. Phys.* **2018**, *20*, 023013.
[3]Singh et al. *Phys. Rev. X* **2022**, *12*, 011040.
[4]Huft et al. *Phys. Rev. A* **2022**, *105*, 063111.
[5]Pause et al. *Optica* **2024**, *11*, 222–226.
[6]Holman et al. *Nature* **2026**, *649*, 859–865.
